# Self-healing of early age cracks in cement-based materials by mineralization of carbonic anhydrase microorganism

**DOI:** 10.3389/fmicb.2015.01225

**Published:** 2015-11-04

**Authors:** Chunxiang Qian, Huaicheng Chen, Lifu Ren, Mian Luo

**Affiliations:** ^1^School of Materials Science and Engineering, Southeast UniversityNanjing, China; ^2^Research Institute of Green Construction Materials, Southeast UniversityNanjing, China

**Keywords:** cement-based materials, bacteria, early age, self-healing, mechanism

## Abstract

This research investigated the self-healing potential of early age cracks in cement-based materials incorporating the bacteria which can produce carbonic anhydrase. Cement-based materials specimens were pre-cracked at the age of 7, 14, 28, 60 days to study the repair ability influenced by cracking time, the width of cracks were between 0.1 and 1.0 mm to study the healing rate influenced by width of cracks. The experimental results indicated that the bacteria showed excellent repairing ability to small cracks formed at early age of 7 days, cracks below 0.4 mm was almost completely closed. The repair effect reduced with the increasing of cracking age. Cracks width influenced self-healing effectiveness significantly. The transportation of CO_2_and Ca^2+^ controlled the self-healing process. The computer simulation analyses revealed the self-healing process and mechanism of microbiologically precipitation induced by bacteria and the depth of precipitated CaCO_3_ could be predicted base on valid Ca^2+^.

## Introduction

With low tensile strength, concrete may take cracks easily during its service life (Bang et al., 2010). Cracking is one of the main factors causing the degradation of concrete durability. Repairing timely or self-healing for cracks may extend the service-life of concrete structures (Wang et al., 2014). Thus, inspection and maintenance techniques for concrete structures have therefore become the focus of interesting attention. However, the traditional repair methods such as cement grouting and maintenance are all difficult and expensive. Under such circumstances, the ability of self-healing cracks in concrete raised wide spread attention.

The phenomenon of self-healing in concrete has been known for many years (Jacobsen and Sellevold, 1996; Edvardsen, 1999; Huang et al., 2013). Precipitation of calcium carbonate has been reported to be the most significant factor influencing the self-healing of concrete (Edvardsen, 1999). Many researchers (Gollapudi et al., 1995; Hill and Sleep, 2002; Rodriguez-Navarro et al., 2003) found that some bacteria could induce or improve the precipitation of CaCO_3_. This phenomenon which was called bio-mineralization existed widespread in nature. Since last decade, Ramakrishnan et al. (1998) published pioneer papers in 1998, bio-mineralization has been developed for the treatment of concrete cracks.

Two main cracks repair mechanisms were proposed in the recent studies. One was proposed by Bang who found one type of bacteria could decompose urea to CO${}_{3}^{2-}$ which can react with Ca^2+^ to form CaCO_3_ in alkaline environment (Stocks-Fisher et al., 1999; Bang et al., 2001; Dhami et al., 2013). This mechanism was applied mainly for passive repair. However, it is very hard to handle the ammonia decomposed from the urea. The other mechanism mainly applying for self-healing was proposed by Jonkers (Jonkers, 2007; Jonkers and Schlangen, 2008; Jonkers and Thijssen, 2010; Jonkers et al., 2010). One type of bacteria which could decompose specific substrate to CaCO_3_ was used in his studies. CO_2_ released in the reaction process which could also trigger CaCO_3_ precipitated in cracks. But the speed of substrate decomposed is slow and the quantity of CO_2_ from substrate is limited.

As some microbes in the nature can conduct the inter-conversion between CO_2_ which in atmosphere and CaCO_3_, Dreybrodt et al. (1998) thought in the H_2_O–CO_2_–CaCO_3_ system, the slow reaction ${\text{HCO}}_{3}^{-}+{\text{H}}^{+}\to {\text{H}}_{2}\text{O}+{\text{CO}}_{2}$ was considered as one of the rate-limiting steps for the precipitation rate of calcite from supersaturated solutions. Carbonic anhydrase (CA) can catalyze the inter-conversion of CO_2_ and HCO${}_{3}^{-}$ to improve the absorption of CO_2_(Heck et al., 1994; Mirjafari et al., 2007). Therefore, CA can aid in the capture of CO_2_ and the precipitation of CaCO_3_, following the equations by the research of Li et al. (2011):

$$\begin{array}{l} {\text{ H}}_{2}\text{O}+{\text{CO}}_{2}\to {\text{HCO}}_{3}^{-}+{\text{H}}^{+}\\ {\text{Ca}}^{2+}+{2\text{HCO}}_{3}^{-}\to {\text{CaCO}}_{3}+{\text{H}}^{+}+{\text{HCO}}_{3}^{-}\to {\text{CaCO}}_{3}\\ \;+{\text{H}}_{2}\text{O}+{\text{CO}}_{2} \end{array}$$

In this study, one type of bacteria which can produce carbonic anhydrase was incorporated in cement-based materials to repair cracks. Compared to other self-healing in concrete with bacteria (Bang and Ramakrishnan, 2001; Jonkers, 2007; Wang et al., 2012), the new bacteria can promote the inter-conversion between CO_2_ which from atmosphere and CaCO_3_. That means CO_2_ can be transferred to minerals precipitated in cracks when reacting with soluble Ca^2+^ rapidly. The bacteria showed excellent repairing ability to small cracks. The depth of CaCO_3_ precipitated layer was also predicted base on valid Ca^2+^ and practical experiment verified the accuracy of predicted results.

## Materials and methods

### Bacterial strain

**Figure 1:**
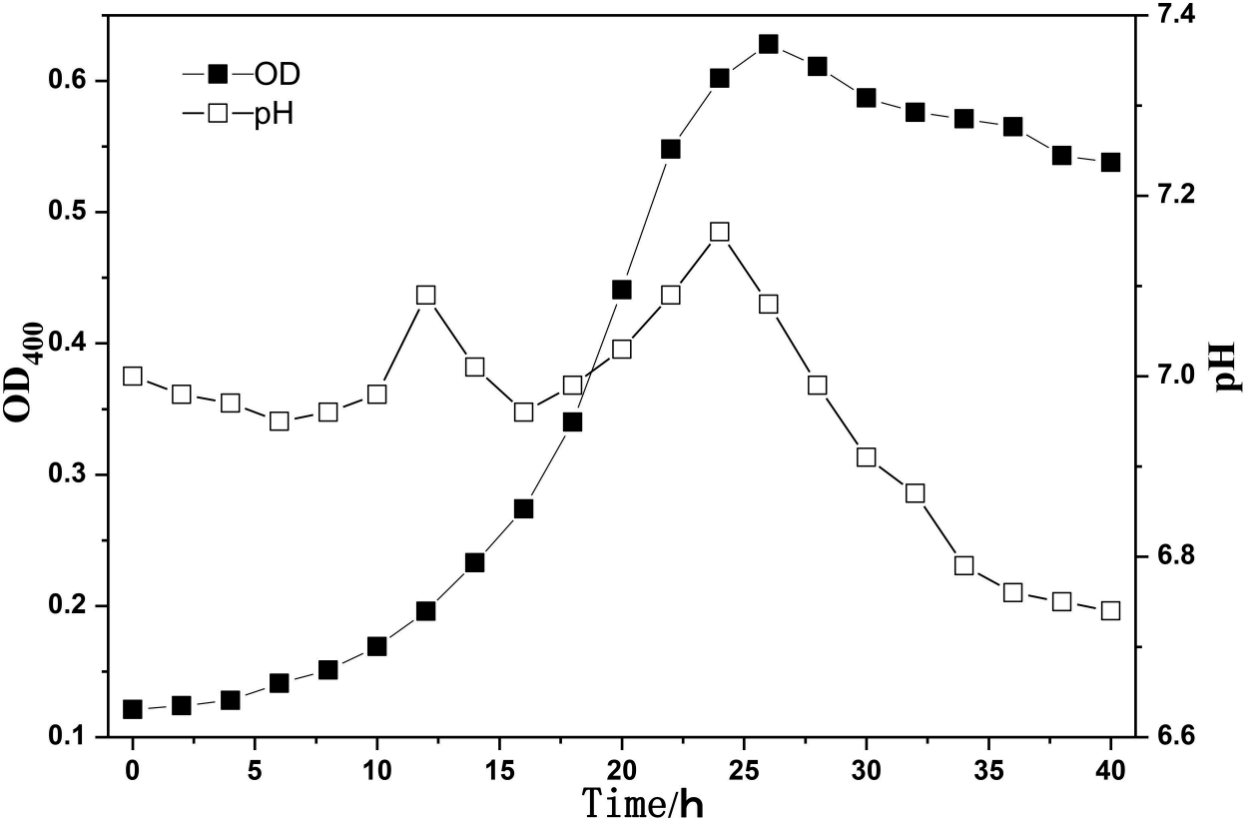
**The growth characteristics of the microorganism**.

*Bacillus mucilaginous L3* (China Center of Industrial Culture Collection, CICC) was used in this study. The growth characteristics of the microorganism is shown in Figure 1.

The results indicated that the best incubation time was about 24 h, and the OD_400_ was 0.8. After incubated for 24 h in specific liquid medium, the fresh bacterial strain were harvested by high-speed (8000 rpm) centrifugation at 4°C for 10 min. Then the bacterial strains were suspended in sterile deionized water. The final concentration of bacterial strain was about 10^9^ cells/mL.

### Preparation of the specimens

Cement paste specimens were used for all analyses in this study. All mixtures were designed with a water to cement ratio (w/c) of 0.40 by using ordinary Portland cement II 42.5. Four series of specimens were made and the composition of each series is shown in Table 1.

**Table 1:** **Composition of the specimens in each series**.

**Group**	**Cement (g)**	**Water (g)**	**Nutrient (g)**	**Bacteria (g)**
G1	1300	520	0	0
G2	1300	507.8	40	0
G3	1300	480	0	40
G4	1300	467.8	40	40

Group G1 are the specimens without any additions. Group G2 are the specimens with nutrient needed for bio-deposition. Group G3 are the specimens only with bacteria. Group G4 are the specimens with nutrient and bacteria at the same times.

Two types of cement specimens, cylinders (φ, 110 mm, H, 45 mm) for testing water permeability and prisms (40 × 40 × 160 mm) for investigating the healing area of cracks, were made. After casting, all molds were put in curing room with a temperature of 20°C and relative humidity of more than 90% for 24 h. The specimens were then de-molded and placed under the standard curing room with a temperature of 20 ± 2°C and relative humidity of 95% until the time of testing.

### Cracks formation and self-healing incubation

**Figure 2:**
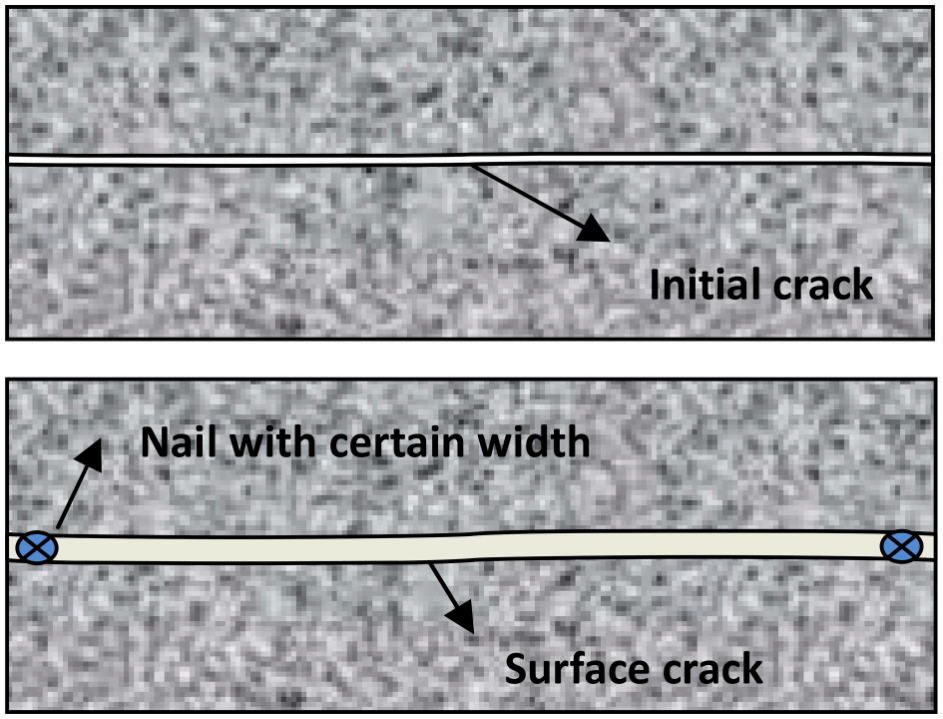
**Crack creation process by embedded method**.

The specimens from Group G1 to Group G4 were taken out from curing room and early age cracks were created after 7 days. The prisms specimens were wrapped with adhesive tape before bending test. The initial cracks were made by a bending load which was loaded to the prisms specimens until the crack break through the cross section. Then nails with different width were embedded into the initial cracks to create the surface cracks with different width. The cracks formation on prism specimens by embedded method was shown in Figure 2. For prism specimens, the compression test (loading speed 0.5–0.8 Mpa/s) was used to make cracks, whose crack width was below 0.3 mm. For crack-healing quantification under different crack width, the crack width was 0.3, 0.4, 0.5, 0.6, 0.7, 0.8, 0.9, and 1.0 mm, respectively. The crack width measured by crack width measuring instrument (Beijing Koncrete Engineering Testing Technology Co., Ltd) which degree of accuracy is 0.01 mm. For studying the self-healing ability influenced by cracking age, the crack width was controlled in the range of 0.4~0.6 mm. To study the repair ability influenced by cracking time, the cracks were created, respectively, after 7, 14, 28, and 60 days.

The treated cracked specimens were immerged in tap water which was exposed to the atmosphere during the whole repair period. Air was pumped into water to keep the adequate supply of CO_2_.

### Characterization methods

**Figure 3:**
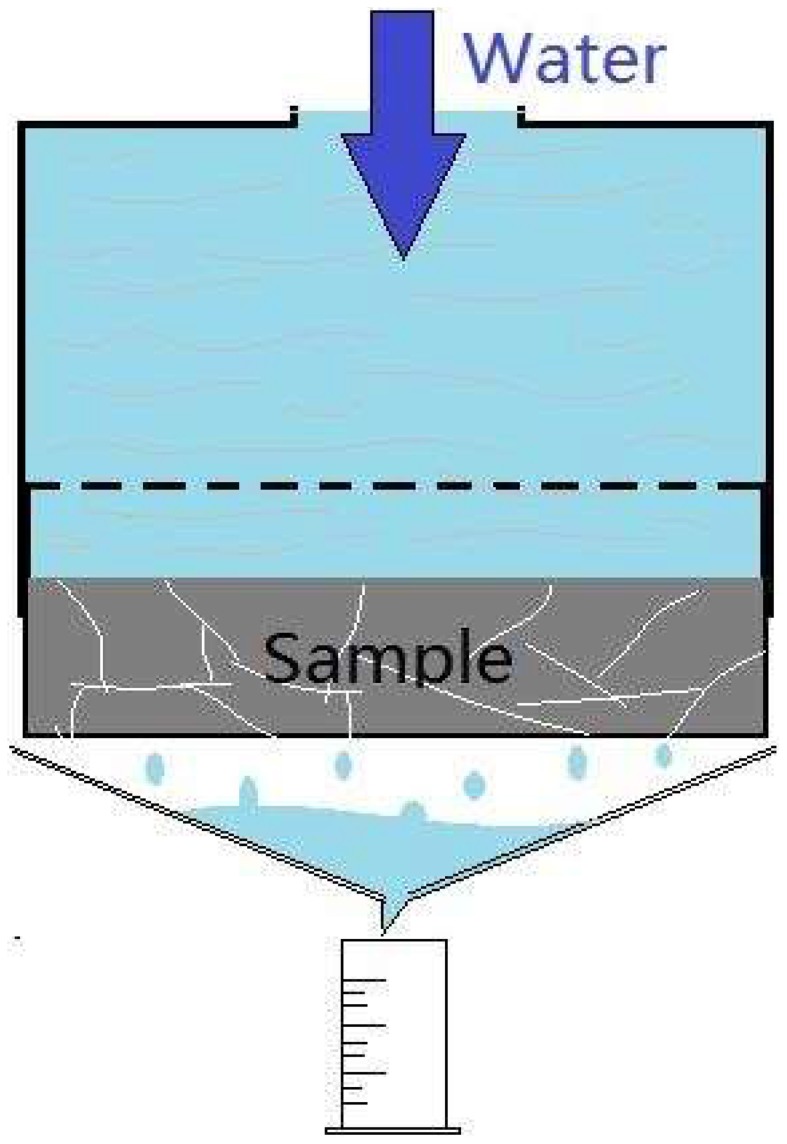
**Schematic diagram of water permeability test**.

Three methods were used to characterize the cracks healing efficiency. The crack healing efficiency of specimens was evaluated by water permeation coefficient according to the method reported with a modification (Wang et al., 2012). The cylinder specimen was casted into PVC mold when molding. PVC mold could be connected to the PVC pipe before test and the joint was sealed tightly to avoid leakage. The water in PVC pipe was kept a fixed height to maintain a constant pressure on the surface of cylinder specimen. The volume of passed water could be measured easily during one period of time. The schematic diagram of water permeability test was show in Figure 3. Permeability coefficient of cylinder specimen before and after healing could be calculated according to Darcy's Law shown in Equation (1). Where k (m/s) is permeability coefficient, Q (m^3^/s) is the amount of water flow, L (m) is the height of specimens, A (m^2^) is the area of section and Δh (m) is head difference.

(1) $$\begin{array}{l} \text{k}=\text{Q}∙\text{L}∕\text{A}∙\text{Δh} \end{array}$$

**Figure 4:**
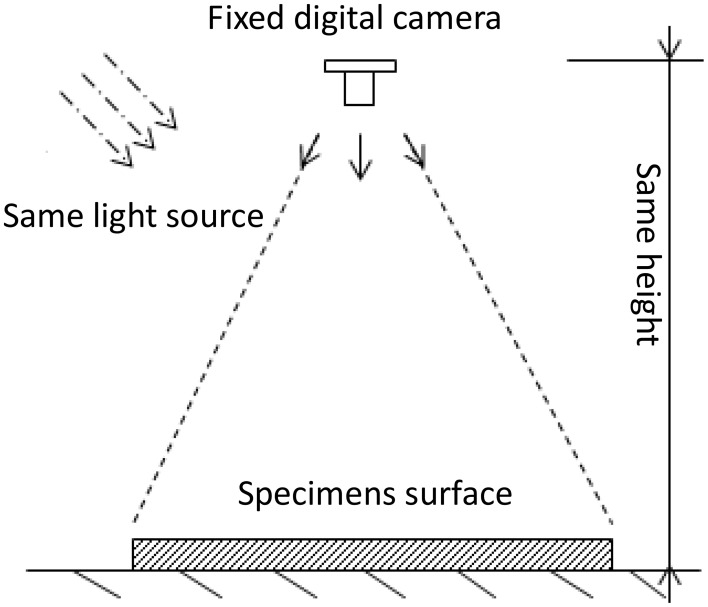
**Schematic diagram of image device**.

Another method to evaluate self-healing was put forward based on image processing. Images for cracks were taken by crack width measuring instrument at the same light source (Figure 4). Image-J was used to achieve the binary photograph and analyze the gray value. Threshold cutting could be achieved easily by setting the gray level threshold of crack at 115 by Image-J. When the image processing of specimen surface cracks at different healing time was carried out, crack areas (pixels) in initial and in different healing time could be counted and the healing efficiency was described by Equation (2). Where α is the area repair rate, A_0_ is the initial crack area, A_t_ is the crack area at healing time. The area repair rate is:

(2) $$\begin{array}{l} \alpha =\frac{{\text{A}}_{0}-{\text{A}}_{\text{t}}}{{\text{A}}_{0}}\times 100\% \end{array}$$

The flexural strength after repaired with different self-healing agent was tested according to the method reported by Li et al. (2013). The three-point flexural loading setup was from MTS Industrial Systems (China) Co., Ltd. The specimens were firstly loaded under three-point flexural configuration and then assembled a whole with tape. Nails were inserted the cracks to control cracks width. After curing certain days in water, the specimens were reloaded as the same testing.

### CO_2_ diffusion in cracked concrete

It was hypothesized that the diffusion of CO_2_ conformed to Fick's law in this study (Equation 3). Where c (kg/m^3^) is the concentration of CO_2_ in concrete, D (m^2^/s) is the diffusion coefficient, t (s) is time.

(3) $$\begin{array}{l} \frac{\partial \text{c}}{\partial \text{t}}=\text{D}\frac{\partial^{2}\text{c}}{\partial {\text{x}}^{2}}+\text{D}\frac{\partial^{2}\text{c}}{\partial {\text{y}}^{2}} \end{array}$$

The CO_2_ diffusion in cracked concrete can be derived by the CO_2_ diffusion in sound concrete. According to the research of Saetta et al. (1995), the diffusion coefficient (D_c_) in sound concrete can be simplified as Equation (4). Where D_c, 0_ is CO_2_ diffusion coefficient in sound concrete curing at 28 days when temperature is 20°C and relative humidity is 65%. D_c, 0_(m^2^/s) can be described as Equation (5) according to 50 dates in papers (Chen, 2007) which were the relationship between D_c, 0_ and compressive strength of concrete. Where f_cuk_ (Mpa) is compressive strength of concrete. F_3_(T) is the temperature factor in Equation (6) and F_4_(H) is the humidity factor in Equation (7). Where K is absolute temperature, RH is relative humidity, RH_0_ = 65%.

(4) $$\begin{array}{l} \text{Dc}={\text{D}}_{\text{c},0}∙{\text{F}}_{3}\left(\text{T}\right)∙{\text{F}}_{4}\left(\text{H}\right) \end{array}$$

(5) $$\begin{array}{l} {\text{D}}_{\text{c},0}=\left(3.60∙{\text{e}{}^{-0.067}}^{{\text{f}}_{\text{cuk}}}\right)\times 10^{-8} \end{array}$$

(6) $$\begin{array}{l} {\text{F}}_{3}\left(\text{T}\right)=0.02\text{K}-4.86 \end{array}$$

(7) $${\text{F}}_{4}(\text{H})={\left[(1-\text{RH})/(1-{\text{RH}}_{0})\right]}^{2.2}$$

**Figure 5:**
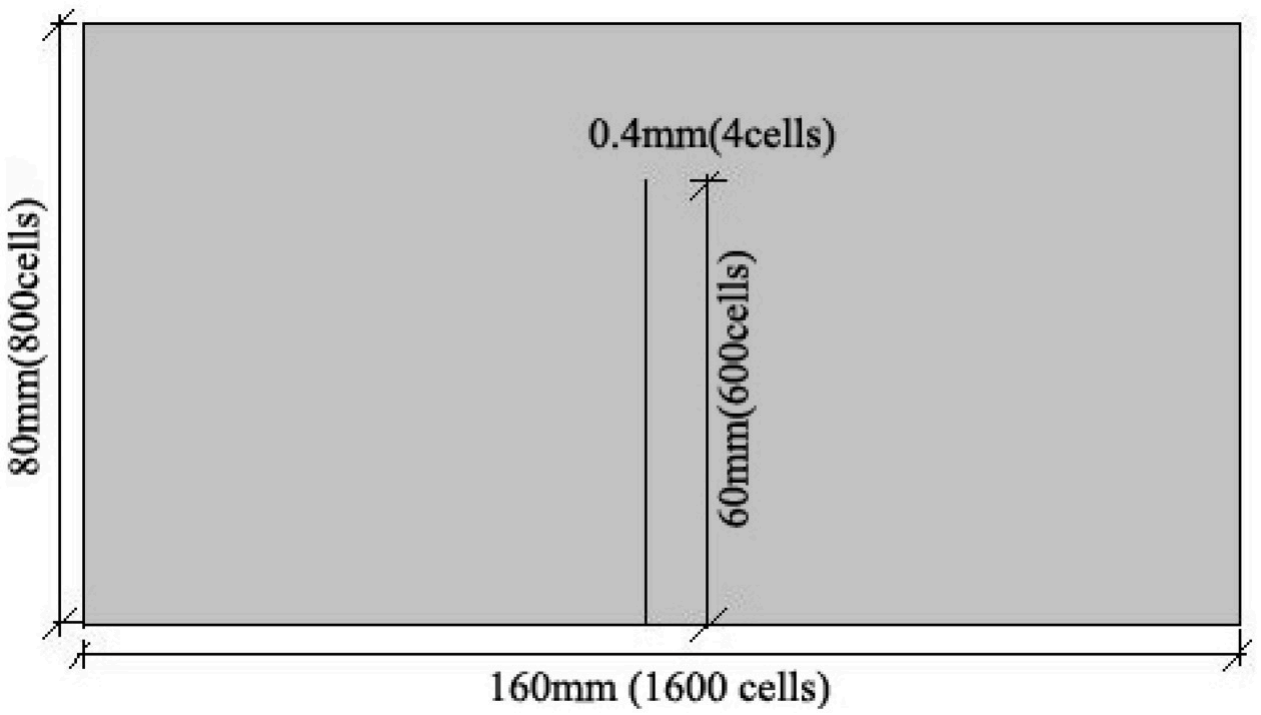
**Schematic diagram of the cracking concrete model**.

The diffusion coefficient of CO_2_ in crack relates to crack width according to the study of Song et al. (2006). In this study, cracking specimens were cured in water. When the crack was filled in water, ${\text{D}}_{{\text{c},\text{H}}_{2}\text{O}}=2.35\times 10^{-6}∙\text{exp}\left(-2119∕\text{T}\right)$ according to the research of Versteeg and van Swaaij (1988). The concentration of CO_2_ on boundary condition is 6.4216 × 10^−4^ kg/m^3^ according to Henry' Law. The model of concrete (16 × 8 cm) is shown in Figure 5. In this model, the size of each mesh is 0.1 × 0.1 mm, the depth of crack is 6 cm and the width of crack is 0.4 mm. The calculation parameters in this model were shown in Table 2, Where D_c_ is the diffusion coefficient of CO_2_ in crack, D_0_ is the diffusion coefficient of CO_2_ in sound concrete and C_0_ is the concentration of CO_2_ on boundary condition.

**Table 2:** **Calculation parameters in numerical simulation**.

**Calculation parameters**		
**D**_c_	**D**_0_	**C**_0_
1.6991 × 10^−9^ m^2^/s	2.6609 × 10^−10^ m^2^/s	6.4216 × 10^−4^ kg/m^3^

### Ca^2+^ dissolving in cracked specimens

Group G2 and Group G3 in Section Preparation of the Specimens were used to study the dissolution of Ca^2+^ in cracked specimens. The prisms specimens were took out after 28 days curing and different crack width (0.2, 0.4, 0.6, 0.8, and 1.0 mm) were created. All surfaces of specimens were sealed with paraffin wax, only cracks were exposed to outside. Then the specimens were immerged in deionized water, the concentration of Ca^2+^ in water was measured by EDTA titration in 3, 7, and 14 days. The specimen (G2) only with nutrient [Ca(NO_3_)_2_] was as a control test.

## Results and discussion

### Crack-healing efficiency of self-healing agent

**Figure 6:**
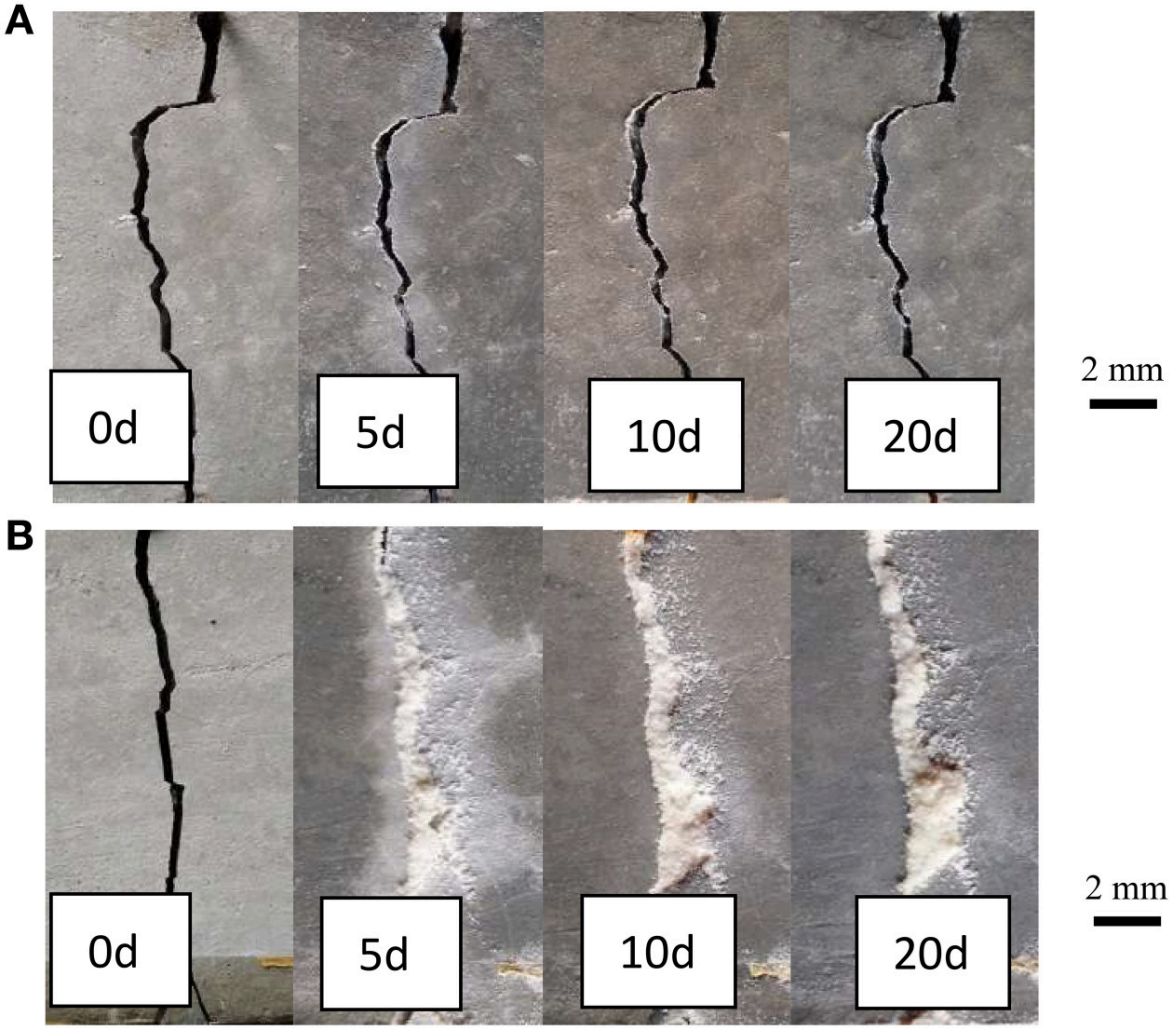
**Surface images of specimens after different healing time. (A)** G1, Specimens without self-healing agent; **(B)** G4, specimens with self-healing agent.

Figure 6 showed the images of the early age cracks during the process of self-healing. Where (Figure 6A) was specimen in Group G1 and (Figure 6B) was specimen in Group G4. It can be seen that crack was almost filled completely after 5 days in Group 4 which cooperated with self-healing agent. But the specimen in Group G1 with no self-healing agent couldn't be healed even curing 20 days.

**Figure 7:**
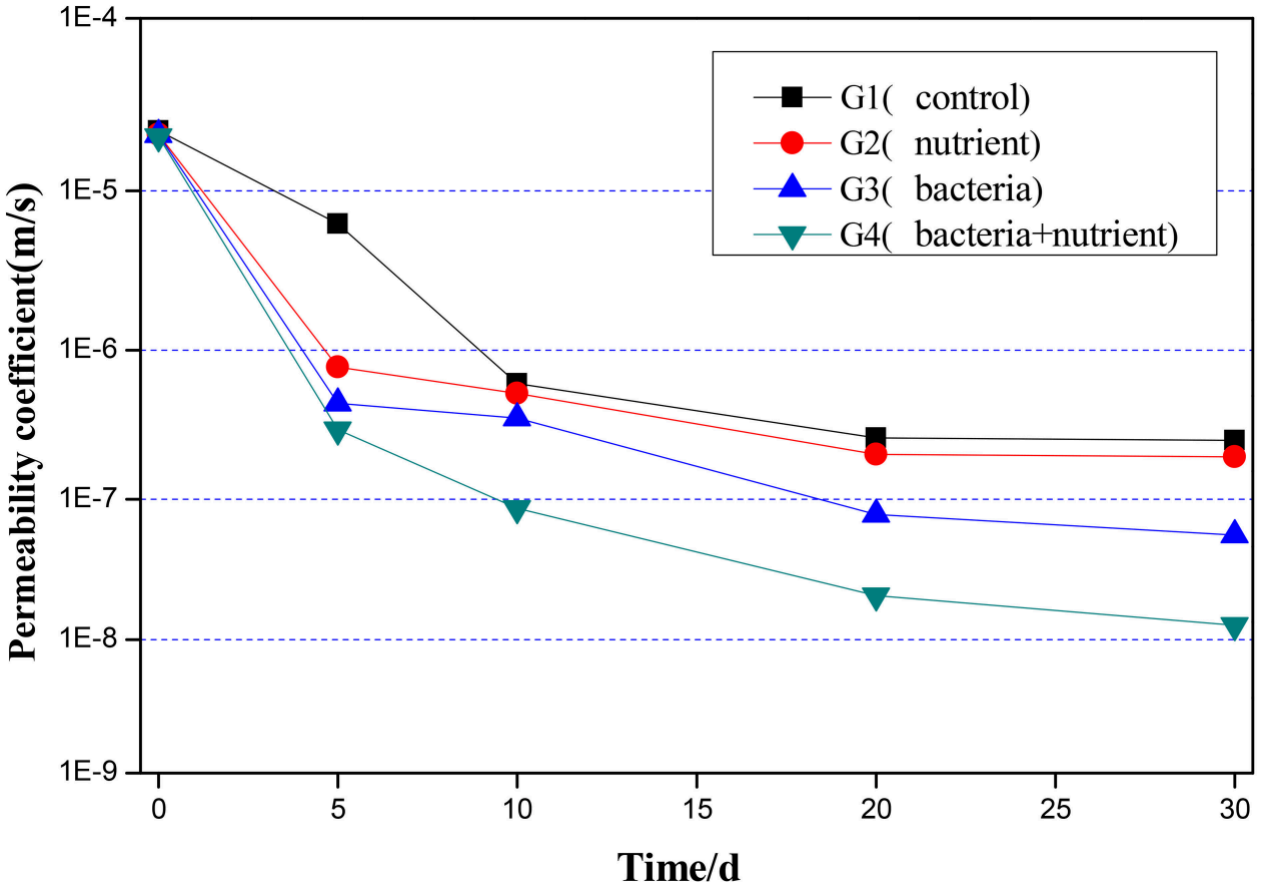
**Water permeability of specimens with different treatment after different healing time**.

Figure 7 showed the permeability coefficient changed over healing time in each group. The initial permeability coefficient of all specimens was about 2.0 × 10^−5^ m/s. The water permeability of specimens in each group decreased after 30 days of immersion in water. The permeability coefficient of specimen in Group G4 declined to 1.28 × 10^−8^ m/s after 30 days, and the phenomenon of water permeability had disappeared. The permeability coefficient of specimen in Group G1 only declined to 2.53 × 10^−7^ m/s after 30 days and the permeability-resistant ability couldn't be improved efficiently during the healing process. The permeability coefficient of specimen in Group G3 only adding bacteria was lower than Group G2 only adding nutrient. It can be concluded that the bacteria could improve CaCO_3_ precipitate to heal the cracks.

**Figure 8:**
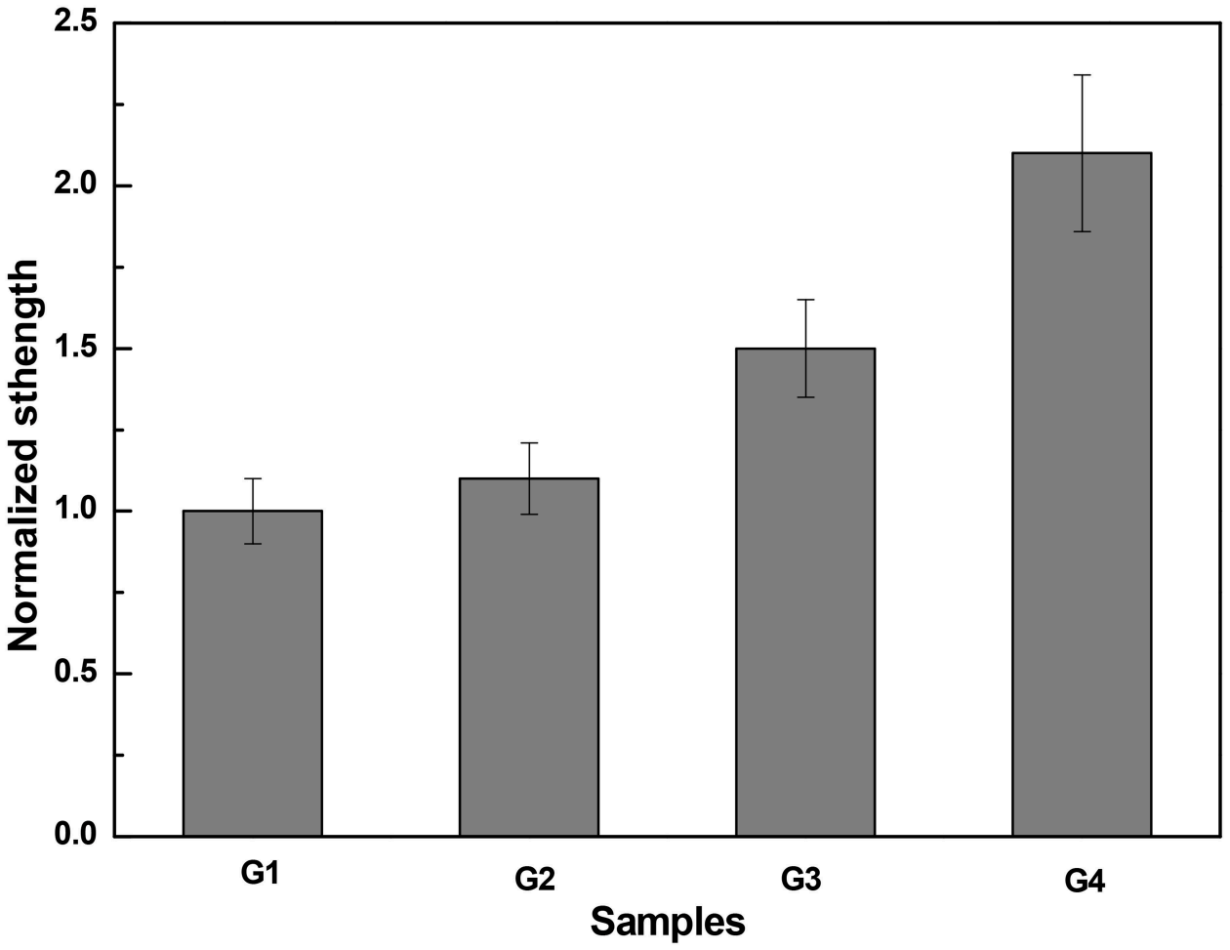
**Influence of different self-healing agent on flexural strength restoration of specimens**.

Moreover, the flexural strength of specimens with different self-healing agent after repaired was tested. The results of the flexural strength for the repaired specimens were shown in Figure 8. Compared to the normalized strength of specimens G1, G2, and G3, the normalized strength of specimens G4 with nutrient and bacteria at the same times reached to 2.1. The results indicated that the flexural strength of specimens repaired with nutrient and bacteria could be increased 40, 90.9, and 110% than other self-healing agent (G3, G2, and G1), respectively.

### Cracking-healing quantification under different crack width

**Figure 9:**
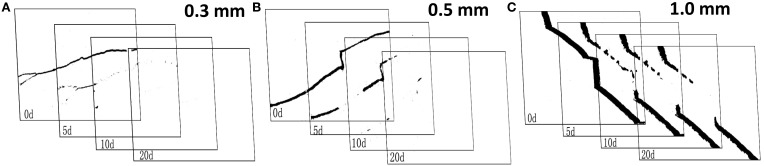
**The binary images of different width crack after different healing time**.

Figure 9 showed the binary images of different width crack which processed by Image-J after different healing stages. The area repair rate of different width cracks was different. The crack with a width of 0.3 mm was nearly closed after 5 days of immersion in tap water. While the crack with a width of 0.5 mm could be healed fully after 10 days. When crack width increased to 1.0 mm, it couldn't be healed well even after 20 days.

**Figure 10:**
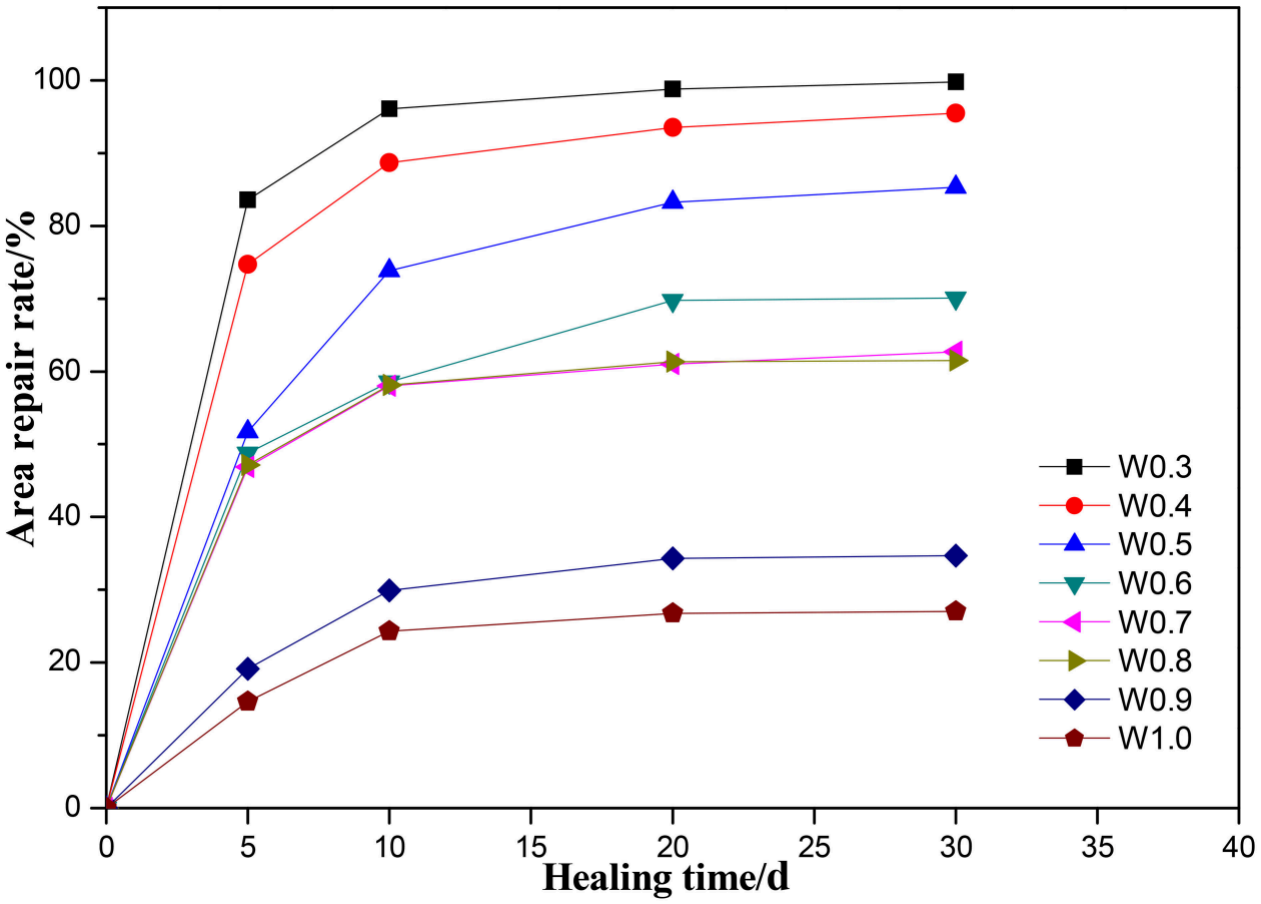
**The average area repair rate of specimens with different crack width**.

The area repair rate of specimens with different crack width after different healing time was calculated based on Equation (2) as shown in Figure 10. It can be seen that the crack was more difficult to repair with the increase of average crack width and the self-healing efficiency of microbial agent declined. Excellent healing effect could be got for the cracks whose average width was below 0.4 mm, the average area repair rate reached over 90% after 30 days. For the cracks between 0.5 and 0.8 mm, the average area repair rate ranged from 60 to 80%, and the larger standard deviation showed obvious difference in self-healing efficiency for different cracks. However, the repair ability of microbial self-healing agent was limited for crack width up to 0.9 mm, and the corresponding average area repair rate was lower than 30% after curing 30 days.

When crack occurred in cement paste specimen, CO_2_ in atmosphere went into crack from crack surface and Ca^2+^ immigrated from cement to crack. CO_2_ would be transferred to HCO${}_{3}^{-}$ induced by microbes in crack and finally precipitated CaCO_3_ when combined with Ca^2+^ in the alkaline environment.

It can be explained as follows: Firstly, The CaCO_3_ precipitation appeared in crack surface, then increased gradually, and fully healed the crack below 0.3 mm. More mineral are needed for larger width cracks for completely healed. Secondly, Ca^2+^ provided by self-healing agent escaped outside to water more easily, resulting in the loss of self-healing agent.

### Cracking-healing efficiency under different cracking age

**Figure 11:**
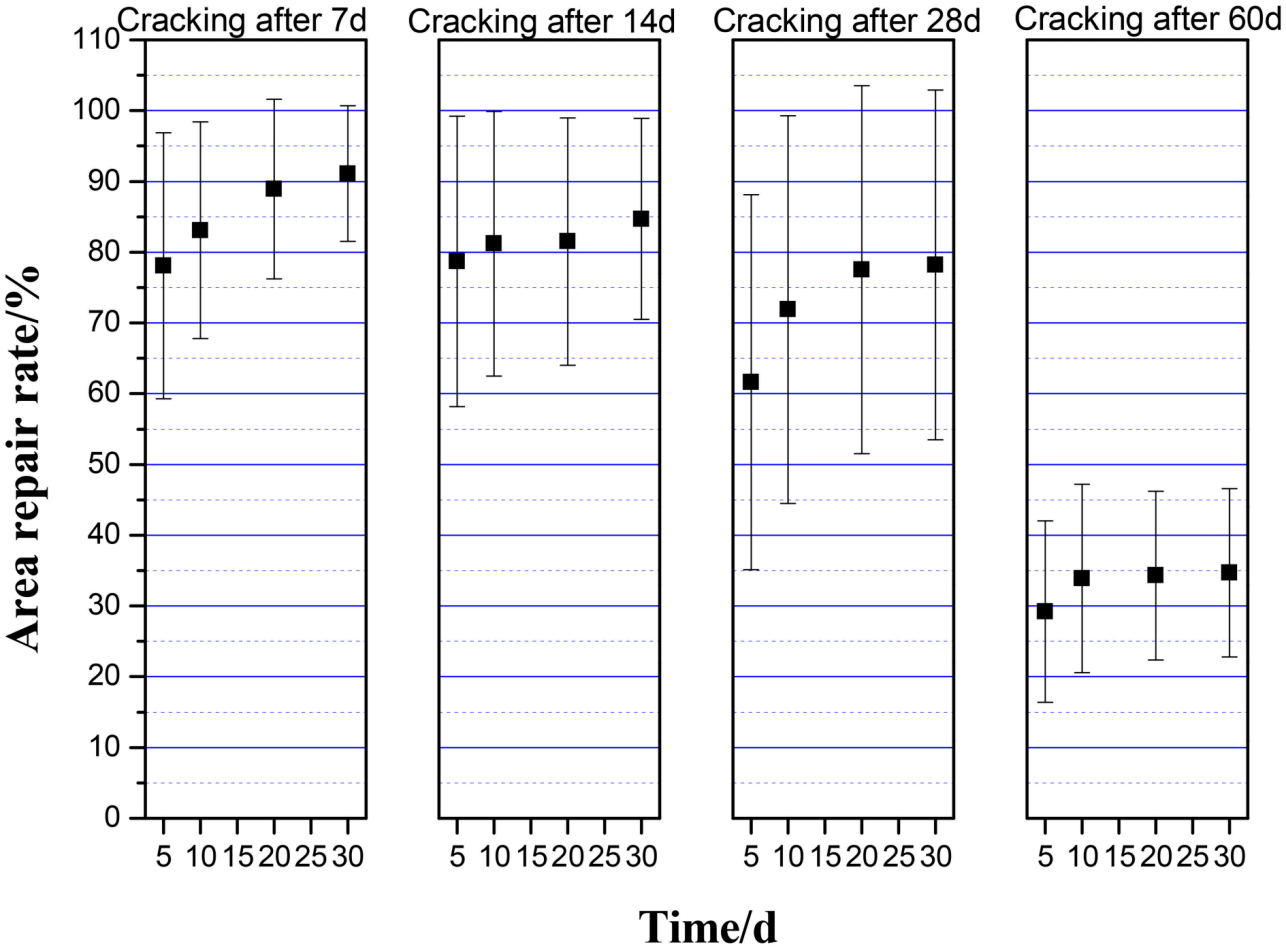
**The average area repair rate of cracks under different cracking age**.

Concrete cracking may occur in any time of service life. As shown in Figure 11, the self-healing effect was excellent for cracking at early age of 7 days. The self-healing efficiency of cracks dropped when cracking age increasing. The area repair rate declined significantly when cracking after 28 days. When the cracking age was more than 60 days, self-healing agent had lost the repair effect. The survival numbers of bacteria in matrix reduced with the increasing of cracking age because of the high alkaline environment in cement-based materials. When the amount of active bacteria reduced to quite small, CaCO_3_ precipitation couldn't be induced. Jonkers et al. (2010) studied the hydration of cement in 28 days would decrease the pores which ranged from 2 to 10 μm greatly in matrix. The living space for bacteria decreased, resulting in death of bacteria. On the other hand, the decrease of pores also made transportation of Ca^2+^ more difficult.

### Numerical simulation of CO_2_ in crack

**Figure 12:**
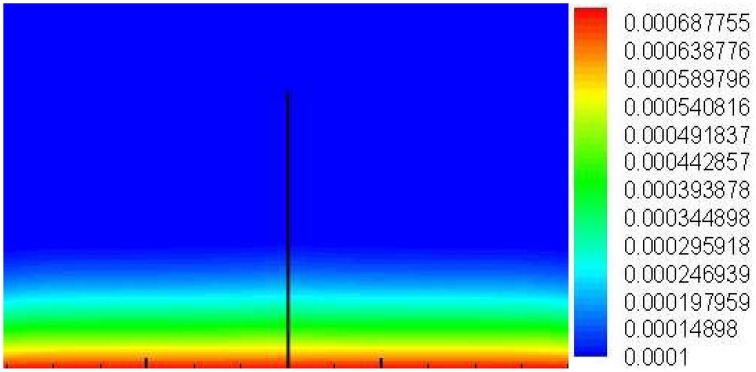
**The concentration of CO_2_ in cracking concrete which immerging in water after 5 days**.

Figure 12 showed the concentration of CO_2_ in concrete when immerging in water after 5 days. It can be seen that the concentration of CO_2_ decreased in depth direction. When the depth was 10 mm, the concentration of CO_2_ declined about 50% compared to crack surface. The concentration on crack surface was highest, leaded to CaCO_3_ precipitated quickly around the crack surface and finally closed the crack. And in depth of crack, the concentration of CO_2_ inside was lower and CO_2_ was insufficient to precipitate CaCO_3_. In other hand, the close of crack surface obstructed the CO_2_ diffusing into crack, further hindered the CaCO_3_ precipitating in the depth direction of crack.

### Predicting CaCO_3_ precipitated depth base on valid Ca^2+^

Table 3 showed the dissolution of Ca^2+^ in Group G2 and Group G4. The amount of dissolute Ca^2+^ in G2 was higher than G4. Ca^2+^in paste immigrated from crack to outside because of the concentration gradient. The bacteria in G4 may impact the amount of dissolute Ca^2+^. Ca^2+^ will cling to bacteria because of its negative cells and the bacteria induce CO_2_ transferring to HCO${}_{3}^{-}$ quickly on crack surface, further combining with Ca^2+^. The difference of the amount of dissolute Ca^2+^ between G2 and G4 was called valid Ca^2+^ which reflected the effect of CaCO_3_ precipitated induced by bacteria. The valid Ca^2+^ meant the part of Ca^2+^ which reacted with HCO${}_{3}^{-}$ then transferred to CaCO_3_.

**Table 3:** **The amount of dissolute Ca^2+^ under different width crack in different group**.

**Time**	**Group**	**Ca**^**2+**^ × **10**^**−4**^ **mol**				
		**0.2 mm**	**0.4 mm**	**0.6 mm**	**0.8 mm**	**1.0 mm**
3 d	G2	1.086	1.925	2.835	3.417	4.012
	G4	0.658	1.342	2.371	3.016	3.656
7 d	G2	2.230	3.453	4.687	5.035	6.391
	G4	1.685	2.835	4.151	4.542	5.993
14 d	G2	3.836	6.695	7.628	7.754	8.333
	G4	2.907	5.975	7.025	7.217	7.897

The valid Ca^2+^ in different crack width was shown in Table 4. It can be concluded that larger crack was benefit to the dissolution of Ca^2+^, but the amount of valid Ca^2+^ was lower in larger crack. Ca^2+^ escaped outside more easily in large crack, but the capture effect of Ca^2+^ by bacteria was limited. The loss of Ca^2+^ which escaped outside to water decreased the amount of valid Ca^2+^. It also can be seen that the amount of valid Ca^2+^ dissolute in 3 days contributed more than 70% to total valid Ca^2+^ dissolute in 14 days except crack width was 0.2 mm. The results indicated that the crack healing occurred mainly at early age. At this period of time, bacteria had high activity to transfer CO_2_ and much HCO${}_{3}^{-}$ could participate in reaction. Crack surface became narrow gradually and finally closed with the increase of healing time. At the late age, the closing of crack surface made the dissolution of Ca^2+^ inside more difficult.

**Table 4:** **The valid Ca^2+^ under different width crack in different group**.

**Time**		**Ca**^**2+**^ × **10**^**−4**^ **mol**				
		**0.2 mm**	**0.4 mm**	**0.6 mm**	**0.8 mm**	**1.0 mm**
3 d	Valid Ca^2+^	0.428	0.583	0.464	0.401	0.356
	Proportion%	46.1	81.0	76.9	74.7	81.7
7 d	Valid Ca^2+^	0.545	0.618	0.536	0.493	0.398
	Proportion%	58.7	85.8	88.9	91.8	91.3
14 d	Valid Ca^2+^	0.929	0.72	0.603	0.537	0.436
	Proportion%	100	100	100	100	100

**Figure 13:**
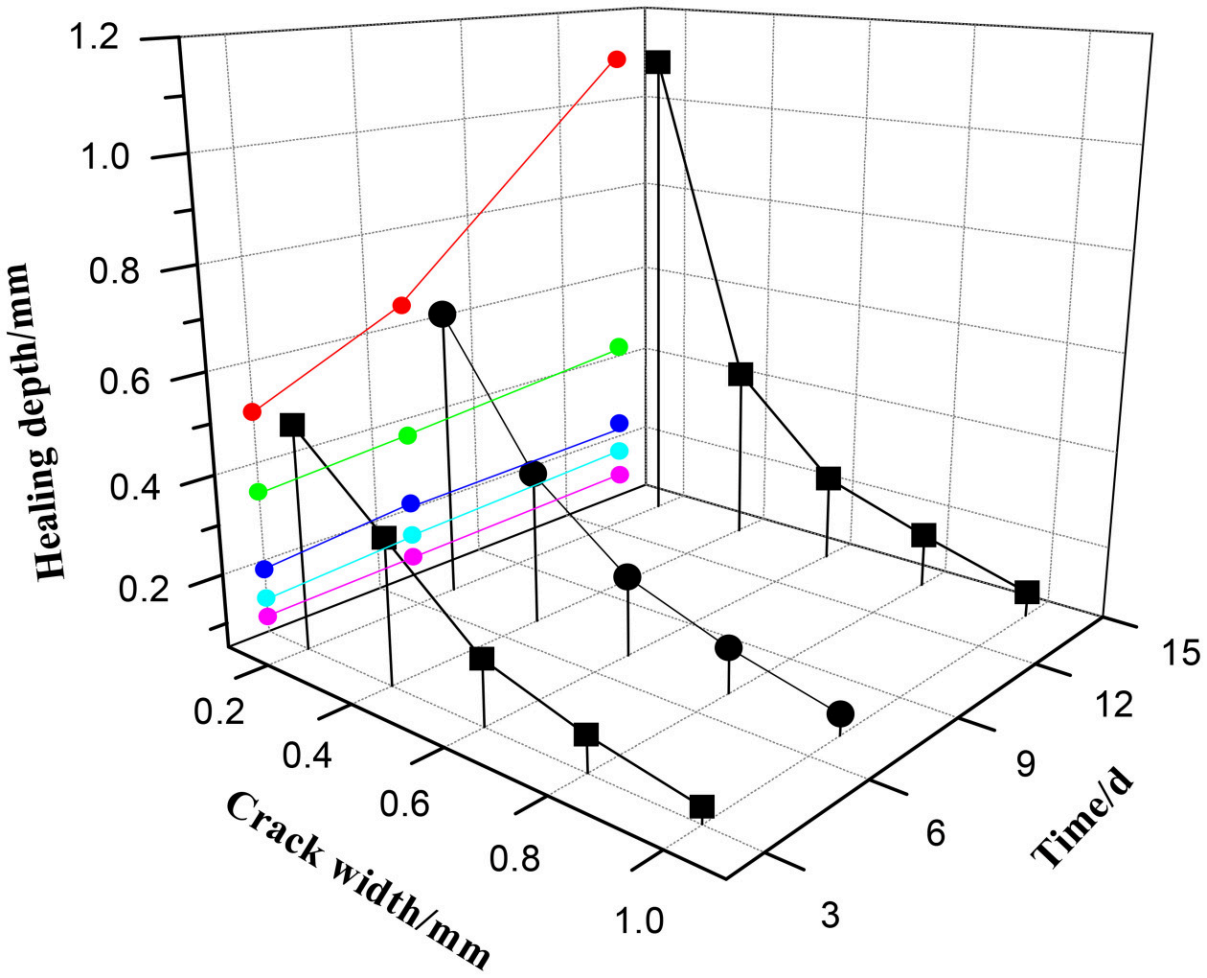
**The predicted healing depth under different crack width after different healing time**.

The depth of precipitated CaCO_3_ could be predicted base on valid Ca^2+^ shown in Figure 13. When crack width was 0.2 mm, the healing depth in crack was, respectively, 0.5, 0.64, and 1.09 mm after 3, 7, and 14 days. The depth of precipitated CaCO_3_ decreased with the increasing of crack width. When crack width was 1.0 mm, the maximum depth of precipitated CaCO_3_ was only 0.10 mm after 14 days.

### Verification of the depth of precipitated CaCO_3_

**Figure 14:**
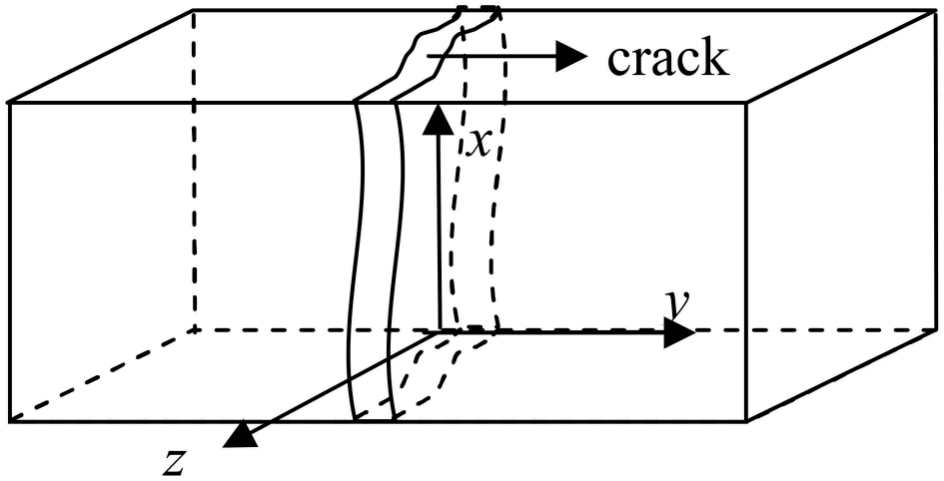
**Schematic diagram of cracking specimens**.

In practical experiment, prisms specimens with self-healing agent were used. The crack (0.2, 0.4, 0.6, 0.8, and 1.0 mm) was made after 7 days curing. Then specimens with crack were immerged into tape water which exposed to atmosphere to 14 days. Figure 14 is the illustration image of cracking specimen.

**Figure 15:**
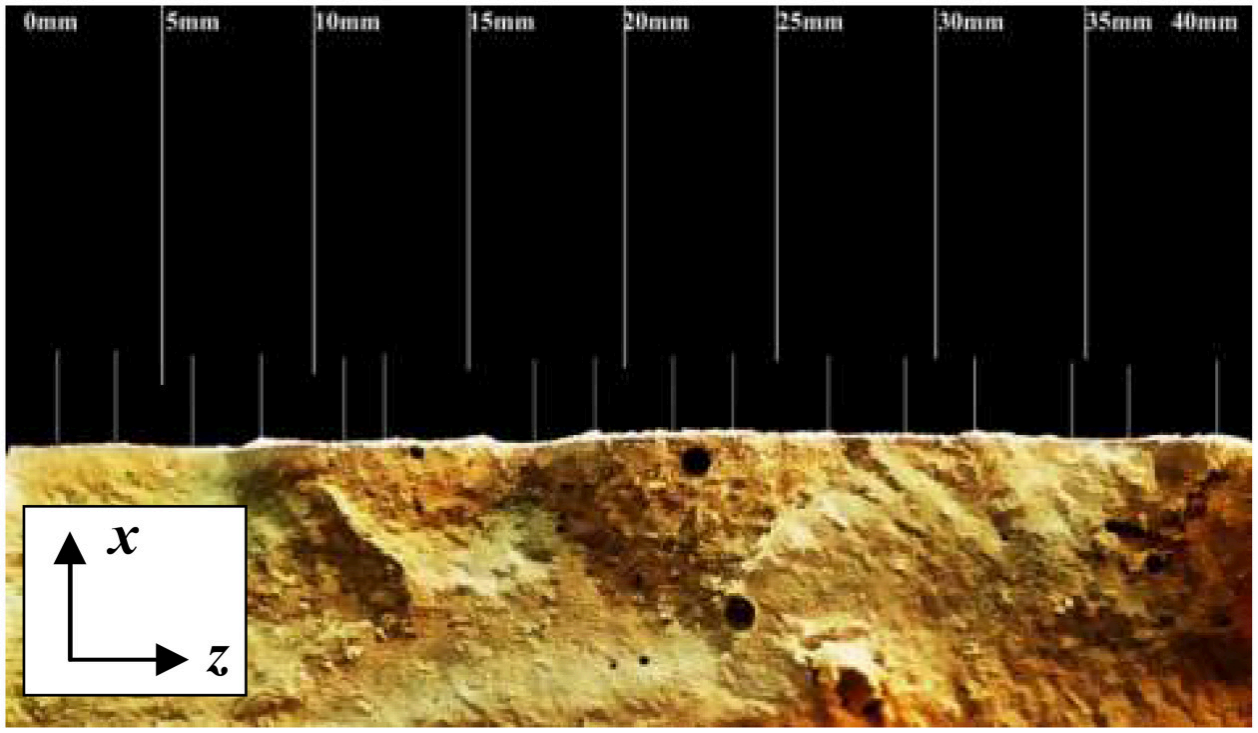
**The image of specimen's fracture section**.

Images of fracture section could be got by digital camera shown in Figure 15. It can be seen that a layer of white sediments precipitated on fracture section. The depth of precipitated CaCO_3_ could be measured by ruler tool. White lines were the measurement position of precipitated depth. Finally the average precipitated depth was calculated to represent the CaCO_3_ precipitated depth.

**Figure 16:**
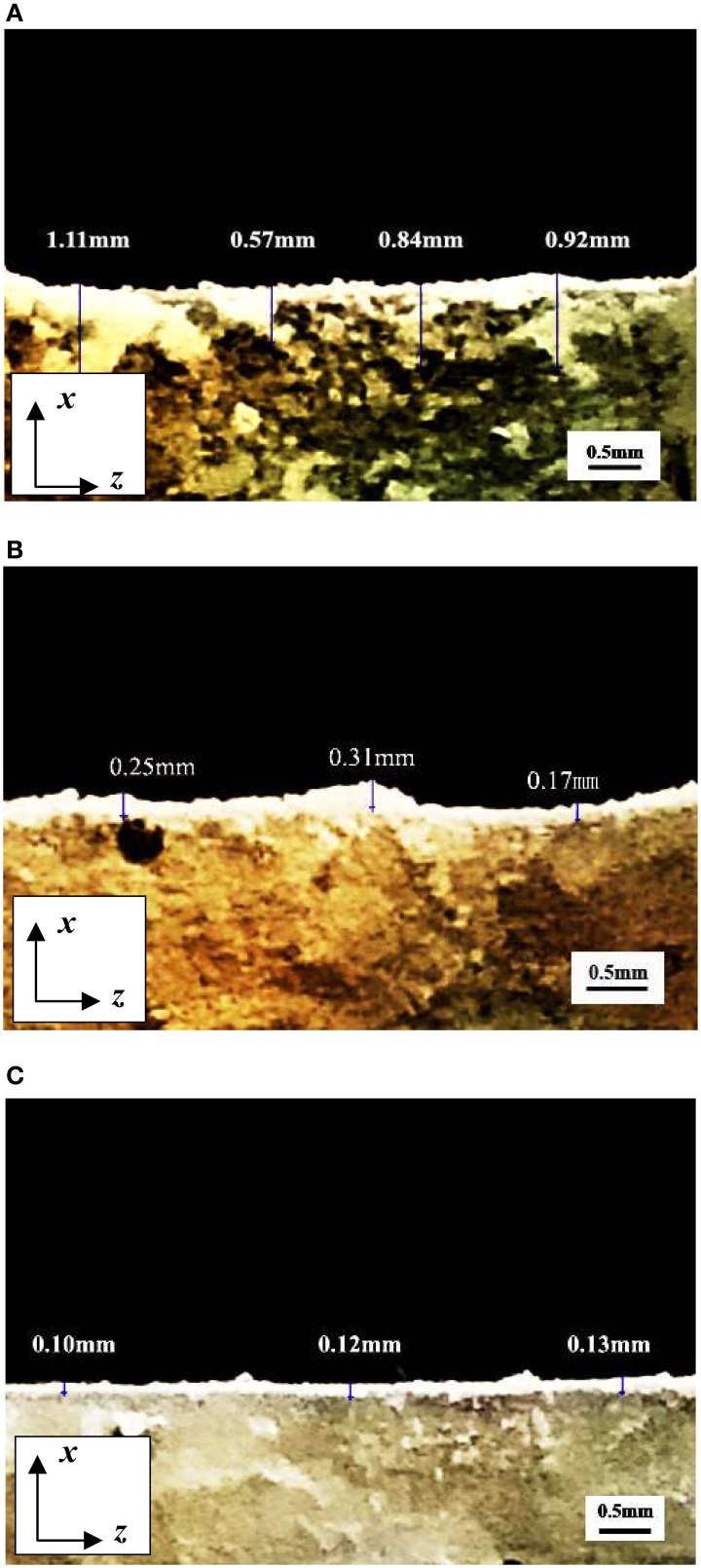
**The CaCO_3_ precipitated in depth direction of fracture section under different crack width**. **(A)** Crack width was 0.2 mm; **(B)** Crack width was 0.6 mm; **(C)** Crack width was 1.0 mm.

Figure 16 showed the CaCO_3_ precipitated in depth direction of fracture section under different crack width. The amount of precipitated CaCO_3_ decreased with the increase of depth and the deposited layer had irregular boundary when crack width was 0.2 mm. The maximum depth of deposited layer was 1.11 mm. When crack width was 0.6 mm, CaCO_3_ mainly precipitated on crack surface. There were no obvious CaCO_3_ found when depth exceeded 0.3 mm. When crack width was 1.0 mm, the depth of precipitated CaCO_3_ layer was only about 0.1 mm.

The comparison between predicted result and experimental result of CaCO_3_ precipitated depth was shown in Table 5. It can be seen that the predicted depth of precipitated CaCO_3_ fit well with the practical experimental results when crack width was larger than 0.2 mm. This result indicted that predicted depth of precipitated CaCO_3_ base on valid Ca^2+^ was accurate.

**Table 5:** **The predicted and experimental result of CaCO_3_ precipitated depth under different crack width**.

**Time**		**Crack width/mm**				
		**0.2**	**0.4**	**0.6**	**0.8**	**1.0**
14 d	Predicted result/mm	1.09	0.42	0.23	0.16	0.10
	Experimental result/mm	0.81	0.42	0.20	0.12	0.11

## Conclusion

The results showed that the bacteria showed excellent repairing ability to small cracks and the self-healing agent can be applied for self-healing of early age cracks in cement-based material. However, the self-healing capacity depended on many factors. The cracks of early age with small width (below 0.3 mm) could be almost fully filled after cured in water.

The diffusion coefficient of CO_2_ in crack relates to crack width was also studied. CO_2_ only focused on the surface of crack whose depth was below 1.0 mm. The highest concentration of CO_2_ on surface leaded to CaCO_3_ precipitated quickly and finally closed the crack, further hindered CO_2_ diffused into crack. And in depth of crack, the concentration of CO_2_ inside was lower and CO_2_ was insufficient to precipitate CaCO_3_.

The depth of CaCO_3_ precipitated layer was predicted base on valid Ca^2+^ in this study and practical experiment verified the accuracy of predicted results. The depth of precipitated CaCO_3_ declined with the increasing of crack width, which coincided with the self-healing efficiency under different cracks width. A certain amount of CaCO_3_ precipitated on the surface of cracks, finally closed the crack surface, and obstructed the self-healing in depth of crack.

### Conflict of interest statement

The authors declare that the research was conducted in the absence of any commercial or financial relationships that could be construed as a potential conflict of interest.
